# Fractal Scaling of Particle Size Distribution and Relationships with Topsoil Properties Affected by Biological Soil Crusts

**DOI:** 10.1371/journal.pone.0088559

**Published:** 2014-02-07

**Authors:** Guang-Lei Gao, Guo-Dong Ding, Bin Wu, Yu-Qing Zhang, Shu-Gao Qin, Yuan-Yuan Zhao, Yan-Feng Bao, Yun-Dong Liu, Li Wan, Ji-Feng Deng

**Affiliations:** 1 College of Soil & Water Conservation, Beijing Forestry University, Beijing, China; 2 Yanchi Research Station, Yanchi, China; University of Jaén, Spain

## Abstract

**Background:**

Biological soil crusts are common components of desert ecosystem; they cover ground surface and interact with topsoil that contribute to desertification control and degraded land restoration in arid and semiarid regions.

**Methodology/Principal Findings:**

To distinguish the changes in topsoil affected by biological soil crusts, we compared topsoil properties across three types of successional biological soil crusts (algae, lichens, and mosses crust), as well as the referenced sandland in the Mu Us Desert, Northern China. Relationships between fractal dimensions of soil particle size distribution and selected soil properties were discussed as well. The results indicated that biological soil crusts had significant positive effects on soil physical structure (*P*<0.05); and soil organic carbon and nutrients showed an upward trend across the successional stages of biological soil crusts. Fractal dimensions ranged from 2.1477 to 2.3032, and significantly linear correlated with selected soil properties (R^2^ = 0.494∼0.955, *P*<0.01).

**Conclusions/Significance:**

Biological soil crusts cause an important increase in soil fertility, and are beneficial to sand fixation, although the process is rather slow. Fractal dimension proves to be a sensitive and useful index for quantifying changes in soil properties that additionally implies desertification. This study will be essential to provide a firm basis for future policy-making on optimal solutions regarding desertification control and assessment, as well as degraded ecosystem restoration in arid and semiarid regions.

## Introduction

Biological soil crusts (BSCs) refer to the cohesive, thin, horizontal layer of soil surface created by soil crust organisms composed of algae, lichens, and mosses, which are widespread throughout the world [1], [2]. In the last few decades, scholars and wildlife managers have become well aware of their existences and roles in ecological processes. Generally, there have been several comprehensive reviews on BSCs including the classic papers by Evans and Johansen [3], Belnap [1] and Bowker [4], as well as the monographs by Belnap and Lange [5], which synthesized large scale and long-term studies. Although there was large variability in types and numbers of BSCs due to the differences in climate, physiognomy, soil, and vegetations, these studies clearly presented that BSCs play an increasingly important role in ecosystem processes and biogeochemical cycles [2], [3], [6].

Unique among all ecosystems, BSCs in deserts not only engage in multitudinous ecological processes, but also serve as the impetus of the habitation of plants, animals, and fragile physical environments [7]–[9]. These biological communities cover the open space between higher plants [5], and dominate the harshest habitats while enduring drought, cold, soil salinity, strong wind, high temperature and radiation in arid and semiarid regions [10]. In some extreme arid deserts, BSCs are the only plants that can survive [11].

Structures and functions of BSCs in deserts have been more thoroughly investigated recently. The first efforts mostly focused on assortment, morphological characteristics and distribution of BSCs under different environmental conditions. Phylogenetic and morphological diversity and microstructure were commonly distinguished [11]–[14], and spatial distribution was mapped [15]–[17]. BSCs potentially contribute to variability in the soil hydrological cycles in arid and semiarid land and should be considered in the development of hydrologic models [18]–[21]. In general, BSCs can determine the amount, position, and time of water infiltration into desert soil, and their characteristics affect the water distribution of soil profiles [22], especially in significantly increasing topsoil water [23]–[26]. Currently, the interactions between BSCs, soil erosion, and desertification were commonly investigated [27]. BSCs protect sandland surfaces against wind erosion and stabilize flowing sand dunes [28]–[30]. Physical, algae and moss crusts were found in different developmental stages of sand dunes [29]. BSCs perform several functions in carbon sequestration and nitrogen fixation, which has drawn increasing attention from scientists, especially under the conditions of global climate change. [31]. There is also a wealth of literature regarding the effects of BSCs on vegetation succession [32]–[34], and physiological responses of BSCs to variations in environmental conditions [35]–[37].

Studies regarding BSCs are numerous and have led to a much greater understanding of their place in ecosystems; however, BSCs have not been explored in full as of yet. More information is needed regarding details of the mutual relationships of BSCs and soil properties. Especially, little is known about an effective index to quantify the effects on soil properties affected by BSCs in the desertification processes. In recent decades, the possibility of characterizing soil particle size distribution (PSD) using fractal theory has been explored [38]. Significant linear correlations have been observed between fractal dimensions and various soil properties. This offers the possibility of quantifying and integrating information on soil biological, chemical, and physical characteristics measured on different spatial scales [27]. In this study, we hypothesized that the variations in topsoil properties are largely consequences of BSCs establishment and succession. Therefore, changes in topsoil properties were documented across three types of successional BSCs (algae, lichens, and mosses crust), as well as in the sandland in the Mu Us Desert, Northern China. The objectives of this study were: (1) to examine the changes in topsoil properties affected by BSCs; (2) to explore the possibility that fractal dimension (*D*) of soil PSD can be used as a practical index for quantifying variations in soil properties and the implications of desertification.

## Materials and Methods

### Study Area

**Figure 1:**
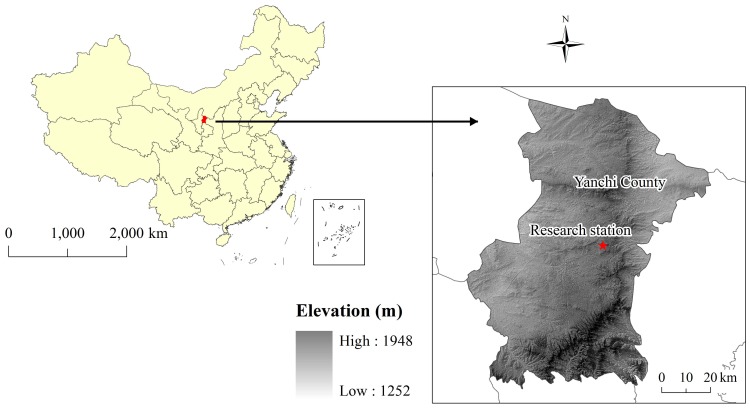
Location of the study area.

The study area is located in Yanchi County (37° 04′ −38° 10′ N, 106° 30′−107° 47′ E; 1400–1800 m a.s.l.) in the central part of southern rim area of the Mu Us Desert in the eastern part of Ningxia Hui Autonomous Region, P. R. China (Figure 1). Yanchi County is a typical transitional zone; the terrain changes from the Loess Plateau (South) to the Ordos Plateau (North) [39]. The specific physical environments make the land quite usable and there are diverse natural resources, but it is a fragile ecological environment.

The study area has a continent-typical warm monsoon climate in a temperate zone. The average annual temperature is 7.8°C. The winter-summer and diurnal-nocturnal differences in air temperature are significantly high, with an annual range in temperature of greater than 28 and 20°C, respectively. The average annual precipitation is 292 mm with 62% occurring between July and September. The average annual evaporation is 2024 mm, which is dramatically higher than annual precipitation. The study area is perennially windy with average annual wind velocity of 2.6 m/s and a prevailing northwest wind direction, which results in frequent and strong wind erosion [40]. The landscape of this area is characterized by sand dunes. The dominated soil type in this area is the arenosols type of quartisamment (U.S. Soil Taxonomy), commonly found in arid and semiarid regions. The typical vegetation of study area is Eurasian Grassland Zone species. The natural vegetation in the study area consists largely of *Salix psammophila*, *Caragana korshinskii*, *Hedysarum scoparium*, *Artemisia ordosica*, *Glycyrrhiza uralensis, Sophora alopecuroides,* and *Cynanchum komanovii*. Additionally, the dominant plantation species include *Salix matsudana*, and *Populus alba*.

The field sampling sites are located in an enclosed area within Zhouzhuangzi Village, Yanchi County, which belongs to the Yanchi Research Station of Beijing Forestry University of the Chinese Terrestrial Ecosystem Research Network (CTERN).

### Study Approach and Experimental Design

#### Soil sampling and crusts identification

The study was conducted from April to October, 2012. The field sampling sites were typical areas of desertification control implemented by fencing after the mid-1990s. In this area, algae, lichens, and mosses crusts exist collectively. To ensure the same parent rock material and similar topography, three random 2 m×2 m sites covering successional BSCs stages were chosen in the open and flat space of sandland with sporadic *Artemisia ordosica* communities in the 1 km range (Table 1). For each BSCs type, three soil sampling profiles were selected at random. Soil samples were collected for two thin layers: the top layer (BSCs layer, the thickness was consistent with BSCs) and the underlying layer (layer under BSCs, 1 cm). Meanwhile, topsoil samples were collected in the referenced uncovered sandland for two thin layers as well: 0–1 cm for the top layer, and 1–2 cm for the underlying layer. Crusts species were identified both macroscopically and microscopically.

**Table 1:** General information of the different BSCs sites.

No.	Type	Color	Thickness (cm)	Coverage (%)	Dominant species
**1**	Algae	Gray	0.37(0.06)	36.7(2.9)	*Microcoleus vaginatus*, *Oscillatoria chlorine*
**2**	Lichens	Brown	0.70(0.12)	53.3(2.9)	*Collema tenax*, *Collema coccophorum*
**3**	Mosses	Green	1.65(0.32)	71.7(5.8)	*Byum argenteum*
**4**	Sandland	–	–	–	–

Values in the parentheses indicates standard error (n = 3).

#### Soil properties analysis

All the soil profile samples were naturally dried in a shaded area in the laboratory; plant stems, small stones, and large animals such as worms were carefully removed. Then, parts of the air-dried soil samples were hand-sieved through 2 and 0.25 mm screens prior to laboratory analysis. To meet the experimental needs, parts of soil samples were ground. The topsoil physicochemical properties were analyzed in the following methods (1) soil organic carbon (SOC) was determined by the potassium dichromate wet combustion method; (2) soil total nitrogen (N_T_) was measured using the micro-Kjeldahl’s method; (3) soil total phosphorus (P_T_) was determined by the Mo-Sb colorimetry method; (4) soil total potassium (K_T_) was measured using the hydrofluoric & perchloric acid (HF-HCLO acid)-flame photometer method; (5) available nitrogen (N_Avi_) was determined by the alkali diffusion method; (6) soil available phosphorus (P_Avi_) was measured using the sodium bicarbonate (NaHCO_3_) digestion-Mo-Sb colorimetry method; (7) soil rapid available potassium (K_Avi_) was determined by the ammonium acetate digestion-flame photometer method [41], [42].

According to the Archimedes Principle, soil bulk density and total porosity were measured using the wax seal method [41], [43]–[44]. Soil bulk density was calculated using Eq. (1); total porosity was subsequently calculated with Eq. (2).
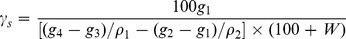
(1)


Where 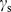 is soil bulk density (g·cm^−3^), same below; *g_1_* is the simple weight (g); *g_2_* is simple weight when completely wrapped by wax (g); *g_3_* is the original reading of electronic balance (g); *g_4_* is reading of electronic balance with the sample (g); 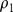 is specific gravity of water, 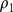 = 1.0 g·cm^−3^; 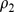 is specific gravity of wax, 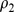 = 0.9 g·cm^−3^; W is water content of sample.
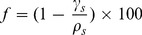
(2)


Where 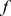 is soil total porosity (%); 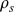 is soil particle density (g·cm^−3^), 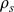 = 2.65 g·cm^−3^.

#### Laser diffraction analysis and soil fractal model

To measure the topsoil particles and fractal characteristics, the unscreened air-dried soil samples were pretreated in an H_2_O_2_ solution (30%, w/w) to destroy any organic matter. And then, the soil aggregates were dispersed by adding sodium hexametaphosphate (NaHMP) and sonicating the samples for 30 seconds [45], [46]. The pretreated soil samples were then analyzed by a laser diffraction technique using Malvern MS 2000 manufactured by Malvern Instruments in Malvern, England with measurement range and margin of error of 0.02–2000 µm and <2%, respectively. Each sample was measured 5 times and the mean value was used. The analysis results of soil PSD were output by U.S. Soil Taxonomy as follow: 0–2, 2–50, 50–100, 100–250, 250–500, 500–1000 and 1000–2000 µm.

Fractal dimension of soil PSD was calculated based on the volume distribution of soil particle size [46], [47]. The equation is expressed as:
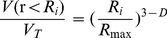
(3)


Where *r* is the soil particle size, *R_i_* is the soil particle size of grade *i*, *R_max_* is the maximum value of soil particle size, *V(r<R_i_)* is the volume of soil particle size less than *R_i_*, *V_T_* is the total volume of soil particles, *D* is the volume-based fractal dimension.

### Data Analysis

All statistical analyses were conducted using SPSS software (Version 17.0). The differences in soil properties and *D* values among the BSCs sites were compared using multiple comparison and one-way analysis of variance (ANOVA) procedures. The LSD test (at *p*<0.05) was used to compare means of soil variables; the results of ANOVA were significant at the level of *p*<0.05. Simple linear regression and correlation analysis were performed to identify the relationships between *D* and the selected soil properties. Pearson correlation coefficient and 2-tailed test were used to distinguish the correlation and significant differences.

## Results

### Soil Bulk Density and Total Porosity under Different BSCs Sites

Variations in soil bulk density were remarkable (Table 2), and significant differences occurred among different BSCs types (*P*<0.05). Uncovered sandland soil had the greatest bulk density (1.71 and 1.69 g·cm^−3^) compared to the BSCs sites, due to severe wind erosion. With the development of BSCs, soil bulk density decreased from algae (1.64 and 1.68 g·cm^−3^) to mosses crust (1.25 and 1.48 g·cm^−3^). Furthermore, soil bulk density within the same type of BSCs increased by 2.4%∼21.2% with increasing soil depth in all sampling sites.

**Table 2:** Variations of bulk density and total porosity for different BSCs.

Item	Layer	BSCs			Sandland
		Algae	Lichens	Mosses	
**Bulk density (g·cm^−3^)**	top	1.64(0.04)a	1.32(0.05)b	1.25(0.01)c	1.71(0.08)d
	underlying	1.68(0.01)ab	1.60(0.02)b	1.48(0.08)c	1.69(0.02)a
**Total porosity (%)**	top	38.29(1.60)a	50.21(1.82)b	52.79(0.52)c	35.58(3.06)d
	underlying	34.64(1.40)a	36.26(1.10)a	42.58(5.57)b	32.40(0.36)a

Values in the parentheses indicates standard error (n = 3). Means with different letter in the same row are significantly different at the 0.05 level (LSD).

Total porosity showed a clear and significant difference in the top layer among different BSCs types; in the underlying layer, significant difference only occurred between mosses crust and other sites (*P*<0.05). However, as shown in Table 2, there was a clear tendency to increase from algae to mosses crust, which was the complete reverse of the contrast to soil bulk density under the different types of BSCs. The lowest total porosity of 35.58% and 32.40% were found in uncovered sandland, and soil total porosity increased successively from algae (38.29% and 34.64%) to mosses crust (52.79% and 42.58%). With the increase in soil depth, total porosity decreased by 3.65%, 13.95% and 10.21% in the same site.

### SOC and Soil Nutrients under Different BSCs Sites

**Figure 2:**
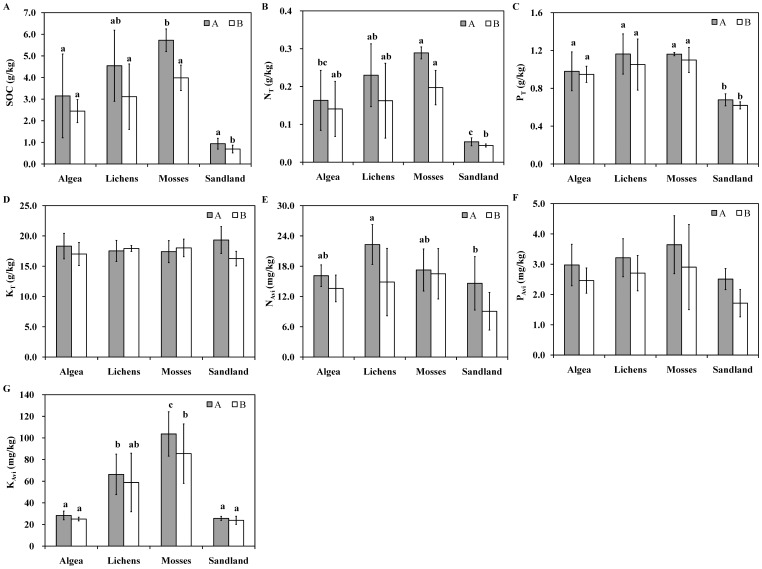
Variations in SOC (A) and soil nutrients (B∼G) under different BSCs. Vertical bars indicate standard errors of means (n = 3). Means with the different letter in the same layers are significantly different at the 0.05 level (LSD).

Accompanying the positive changes in soil physical structure, SOC and soil nutrients increased alike (Figure 2). Comparing with the sandland, SOC was higher in the BSCs sampling sites. Among them, mosses had the highest SOC in both layers (5.72 and 3.98 g·kg^−1^) which differed significantly with the sandland (0.94 and 0.69 g·kg^−1^) (*P*<0.05), but not significantly different from lichens (4.54 and 3.11 g·kg^−1^) and algae (3.15 and 2.45 g·kg^−1^) crusts (*P*>0.05). Further, SOC within the same type of BSCs deceased from the top to underlying layer.

Changes in soil nutrients varied significantly, especially the levels of K_T_ and N_Avi_. However, there was a clear increasing tendency across the three types of successional BSCs, and soil nutrients under BSCs sites were greater than the uncovered sandland. For the content of K_T_, N_Avi_ and P_Avi_, no significant differences were found among the different BSCs types (*P*>0.05). However, there were significant differences in the content of N_T_, P_T_, and K_Avi_ at different sites, especially between the uncovered sandland and mosses crust (*P*<0.05). The spatial variations in soil nutrients in the soil profile deceased from the top to underlying layer as well.

### Particles Size Distribution and Fractal Characteristics under Different BSCs

Table 3 shows the soil PSD in the different soil sampling sites, and sand particles (50–2000 µm) could be considered the dominant soil particle class accounting for more than 80% of the total, approximately. Meanwhile, there was a clear tendency to decrease of sand particle content from algae to mosses crust. Compared with the uncovered sandland, sand particles content decreased by 11.2∼16.4% and 4.9∼12.6%, and sand particles of mosses crust had significant differences with other crust types (*P*<0.05). Clay (0–2 µm) and silt (2–50 µm) contents were much lower than sand particles, especially the clay contents (0.4∼0.7%). However, with the development of BSCs, clay and silt contents increased sharply. Compared with the uncovered sandland, from algae to mosses crust, the clay and silt contents increased by as much as 90.9∼112.1%, 163.9∼241.2% and 27.2∼71.9%, 65.4∼167.2% for the top and underlying layers, respectively. As a result, significant differences occurred between mosses crust and other sites for clay and silt contents (*P*<0.05). Furthermore, sand contents within the same type of BSCs increased from the top to underlying layer, in addition to the decrease in clay and silt contents. In contrast, clay and silt contents of the uncovered sandland were cumulative with the increase in soil depth.

**Table 3:** Variations of soil PSDs for different BSCs.

BSCs	Layer	Soil PSD (%)		
		Clay (0–2 µm)	Silt (2–50 µm)	Sand (50–2000 µm)
**Algae**	top	0.6(0.1)a	16.4(3.7)a	83.0(3.8)a
	underlying	0.5(0.1)ab	11.3(2.0)ab	88.2(2.1)ab
**Lichens**	top	0.7(0.1)a	20.5(7.7)a	78.9(7.8)a
	underlying	0.5(0.2)ab	15.9(7.5)a	83.6(7.6)b
**Mosses**	top	0.7(0.1)a	21.2(0.8)a	78.1(0.9)b
	underlying	0.7(0.1)a	18.3(2.1)a	81.1(2.2)a
**Sandland**	top	0.3(0.1)b	6.2(0.5)b	93.5(0.6)a
	underlying	0.4(0.1)b	6.8(0.9)b	92.8(1.1)b

Values in the parentheses indicates standard error (n = 3). Means with different letter in the same line of the same layer are significantly different at the 0.05 level (LSD).

Volume-based soil fractal dimensions (*D*) were subsequently calculated with Eq. (3) based on the soil PSD data, and the variations in *D* values for the different BSCs are shown in Table 4. Generally, fractal dimensions of soil PSD ranged from 2.1477 to 2.3032; with the development of BSCs, *D* values increased progressively from algae to mosses. Meanwhile, the *D* values of algae, lichens and mosses crust were higher than uncovered sandland in both layers. However, significant differences among the different BSCs types only occurred in the top layer (*P*<0.05). In the underlying layer only mosses significant differed with uncovered sandland (*P*<0.05). Additionally, *D* values observed in the underlying layer were lower than the top layer because the wind-induced fine particles remove surface soil.

**Table 4:** Variations of soil fractal dimensions for different BSCs.

(cm)	BSCs			Sandland
	Algae	Lichens	Mosses	
**top**	2.2786(0.0281)a	2.2904(0.0262)a	2.3032(0.0214)a	2.1477(0.0403)b
**underlying**	2.2291(0.0224)ab	2.2409(0.0575)ab	2.2892(0.0144)a	2.1687(0.0511)b

Values in the parentheses indicates standard error (n = 3). Means with different letter in the same row are significantly different at the 0.05 level (LSD).

### Relationship between Soil Fractal Dimensions and Selected Soil Properties

**Figure 3:**
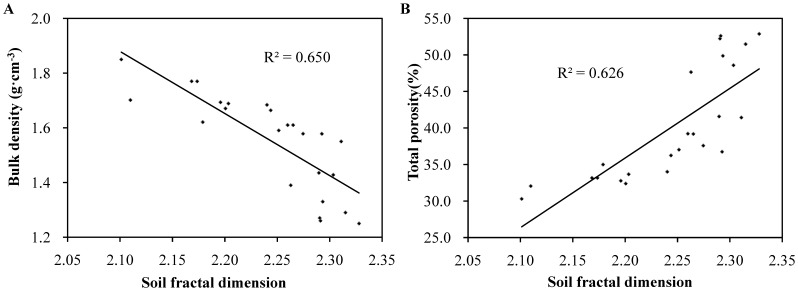
Relationships between *D* values and soil bulk density (A), total porosity (B).

**Figure 4:**
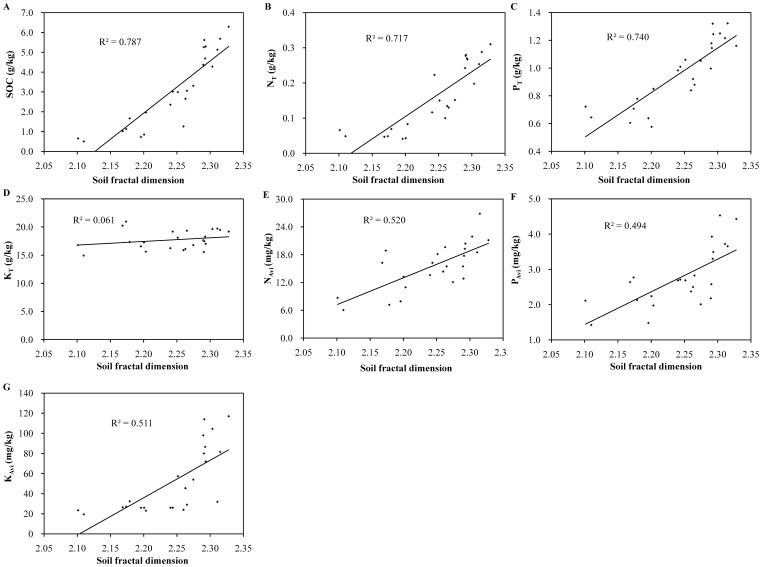
Relationships between D values and selected soil properties (A∼G).

**Figure 5:**
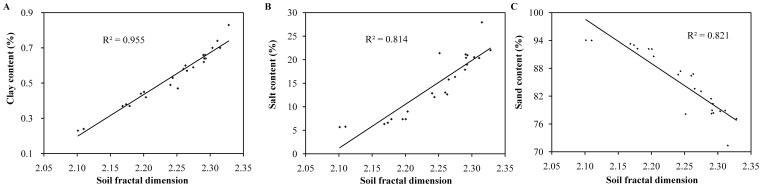
Relationships between *D* values and soil PSD (A∼C).

Linear regression and correlation analysis were applied to identify the relationships between fractal dimensions (*D*) and selected soil properties including soil bulk density, total porosity, SOC, soil nutrients as well as soil PSD (Figure 3–5, Table 5). Results indicated that a significant negative linear correlation between soil bulk density and *D* values was observed with R^2^ = 0.650, *p*<0.01 (Figure 3A). By contrast, Figure 3B showed a positive linear correlation between total porosity and *D* values (R^2^ = 0.626, *p*<0.01). This reverse correlation and the different variations in bulk density and total porosity mutually verified.

**Table 5:** Correlations between soil fractal dimensions (*D*) and selected soil properties.

Soil properties	Correlation coefficients	Soil properties	Correlation coefficients
Bulk density	−0.806**	N_Avi_	0.721**
Total porosity	0.791**	P_Avi_	0.703**
SOC	0.887**	K_Avi_	0.715**
N_T_	0.847**	Clay	0.977**
P_T_	0.860**	Silt	0.902**
K_T_	0.247	Sand	−0.906**

**Correlation is significant at the 0.01 level (2-tailed).

Figure 4 showed the relationship between *D* values and SOC and soil nutrients. The recovery and accumulation of SOC and soil nutrients resulted in increased *D* values. Fractal dimension had considerable significant positive linear correlation with SOC, N_T_, P_T_, N_Avi_, P_Avi_ and K_Avi_ (R^2^ = 0.494∼0.787, *p*<0.01). However, no significant correlation occurred between *D* values and K_T_ (Figure 4D, R ^2^ = 0.060, *p*<0.01). Therefore, soils with more fertilizer have higher fractal dimension values; values of *D* do not appear to be related to K_T_.

In Figure 5, soils with greater clay and silt contents had higher soil fractal dimensions (*D*); soils with a greater amount of sand particles had lower *D* values. *D* values had significant positive linear correlations with clay and silt contents (R^2^ = 0.955 and 0.814, *p*<0.01), and a negative linear correlation with sand particles contents (R^2^ = 0.821, *p*<0.01). Therefore, wind-induced removal of fine particles resulted in decreased *D* values, and *D* values appeared to be closely related to clay particles rather than silt and sand contents.

## Discussion

### Effects of BSCs on Topsoil Properties

In the Mu Us Desert, soil properties can be improved through the addition of plants including the lower plants that introduce BSCs. This information is greatly important for the countries in arid and semiarid regions with a high susceptibility to wind erosion and desertification. BSCs protect sand surface from wind erosion. Erosive force and carriage ability are consumed by BSCs through increasing aerodynamic roughness length and threshold wind velocity [30]. Topsoil loss with airflow under BSCs is much lower than in the uncovered sandland; and the dusts in airstreams are captured by cyanobacteria filaments as well. These processes have positive effects on bulk density decrease and total porosity increase. Moreover, most of the soil photosynthetic productivity and nitrogen fixation in sandland is concentrated within 3 mm of the surface [48]. Therefore, BSCs are meaningful to combat land degradation.

With the presence of BSCs, a significant amount of CO_2_ exchange is observed between BSCs and atmospheric content. CO_2_ is sequestrated and accumulated through a combination of BSCs photosynthesis and respiration. Free chlorophyll in algae and chloroplast in mosses capture CO_2_ through photosynthesis and store carbon in the form of carbohydrates in their tissues. The photosynthetic rate of BSCs has been commonly reported under natural conditions or in the laboratory. However, the values are of great uncertainty, ranging from 0.1∼11.5 μ mol CO^2^ m^−2^·s^−1^ due to the difference in various physical environments and the ability to carbon capture of different crusts species [5]. Synthesizing all these study achievements, Elbert et al [49] obtained a median flux of 16 g·m^−2^·a^−1^ for the net uptake of carbon by BSCs, accordingly, the total carbon sequestration by BCSs in arid and semiarid regions is approximately 1.0 Pg·a^−1^. Although, it is much lower in comparison with other processes of the global carbon cycle, millions tons of carbon was sequestrated by BSCs and stored in soil ecosystem, which helps soils prosper. It is of significant importance for soil environmental restoration, and can’t be ignored of understated.

Nitrogen turnover is as complex as the carbon cycle; it combines nitrogen mineralization, ammonia volatilization, nitrification and denitrification. The presence of dinitrogenase in alga, and bacterium of BSCs play a significant role in this process; these biochemical processes that lead to nutrients fixation [50], [51]. Photosynthesis and azotification are closely interconnected and interdependent. As an important precondition, carbohydrate produced by the BSCs’ photosynthesis drives the azotification of dinitrogenase in alga and bacterium. In exchange, azotification provides much-needed organic nitrogen to sustain BSCs for photosynthesis. Like the photosynthetic rate, the average fluxes of nitrogen fixation are reported in the range of 0.1–10 g·m^−2^·a^−1^; the total nitrogen fixation by BCSs in arid and semiarid region is approximately 30 Tg·a^−1^ [49].

Finally, carbohydrates and organic nitrogen introduced by BSCs are input into soils in various ways. The most important way is through the decomposition of BSCs. At the end of BCS lifecycle, remains decompose through enzymatic activities by the combined action of soil bacterium and enzymes. Furthermore, some algal crusts can secrete polysaccharide into soil to increase carbon pool; similarly, nitrogen micromolecules are released into soil by BSCs as well. Meanwhile, SOC and soil nitrogen accumulation is beneficial to the growth of tracheophyte; successions of vascular plants help soil properties prosper. Additionally, some beetles used BSCs as habitat; these BSCs-feeding beetles are also considered as an important nutrients resource in arid and semiarid regions due to the decomposition of their bodies.

With carbon sequestration and transport at its core, BSCs increase nutrient contents and make soil more fertile. However, unique among other soil nutrients, soil K_T_ is an expectation. In this study, a poor relationship between *D* and K_T_ was observed which corresponded with a lack of differences in K_T_ among the different types of BSCs and differing degrees of desertified soils. In fact, although potassium is one of the most essential macronutrients for plant growth and development, scholars pay little attention on the conversion due to its substantial deposit in soil system of northern China. In nature, potassium of different forms can affect and transform to each other; however the rate of this conversion is rather slow. Among all the forms, potassium mainly exists in a number of inorganic substances, especially the inorganic mineral [52]. These mineral is stable, ion-exchange reactions barely occur with cations in soil solutions, and are instead soaked up by plants. Therefore, soil K_T_ is related to soil mineralogy, and unlikely to be affected by BSCs. Besides, we suspect that K_T_ may lose due to the decrease of pH (from alkalescence to neutral) induced by combination efforts of vegetation recovery and wind erosion.

Variations in soil properties differ between successional stages of BSCs. Complex and evolutional BSCs achieve greater activity and ability as results of diverse community composition in numbers, species, and functions [53]. In uncovered sandland, SOC and soil nutrients are lower than at later successional stages. When algeas crusts develop into lichens, microalgal biomass increases rapidly along with carbon and nitrogen deposition. In mosses crusts, photosynthesis and azotification require a higher environmental threshold; whereas in return mosses crust have stronger resistance to and more efficient abilities of carbon and nitrogen fixation. Therefore, although BSCs create an important increase in soil fertility, there is, at times, no significant difference occurred between sandland and algae, lichens crust. Contrarily, mosses crust significantly differs from sandland, even algae and lichens crusts.

### Soil Fractal Dimension as a Practical Indicator for Desertification

Characterizing soil organization and functions with a single parameter is a question of great interest when monitoring soil degradation and desertification. In former studies, individual fractions (e.g., clay and fine fractions) textual analysis and soil organic matter were the commonly used methods to characterize soil quality. However, soil is an irregular and complex system in which many biological and physical components interact across all space and time scales [54]. Among these approaches, individual fractions always ignore coarse fractions, but emphasize fine particles which are rich in nutrients. Therefore, it cannot provide complete information and cause the waste of soil data. Moreover, it is not ideal to the real soil system, especially the soil in the deserts which coarse particles account for a greater proportion. In addition, although textual analysis provides integrated information, the precision and comparability of data are adversely affected by the descriptive indicator. Furthermore, soil organic carbon is one of the most important indexes in soil quality assessment, but it is not sensitive to environmental change in short-time scale [55]. Therefore, traditional methodologies cannot provide complete information and quantitatively represent fundamental attributes using a practical index. By contrast, fractal measure can make full use of the soil PSD information including clay, silt, and coarse particles data. Fractal dimensions significantly correlated with soil dynamics for long and short time scale. Soil degradation causes an increase in one or more intervals of fractal behavior [56]. Modeling the process of fragmentation both in rocks and soils [57], fractal geometry in soil science have shown that soil exhibits fractal characteristics of irregular shape and self-similar structure [58]. The methodology is of considerable intrinsic interest because it is practical and descriptive, rather than a simplification and simulation [59].

Soil PSD is closely related with soil functions and they are interdependent. It is generally believed that the rich fine fractions (clay and silt) mean fertile, hydrophilic, and biodiversity-rich soil system. Therefore, when wind-induced erosion occurs and soils are lost, we lose far more than just the fractions that they contain, but immeasurable ecological services. Wind erosion not only causes the transport of soil particles, but also nutrient and function losses, and further resulting in the decrease of water-holding capacity, depletion of soil structure and biological properties [27], [60]. Finally, these losses in fine particles affecting by wind-induced erosion cause land degradation, and desertification. In this study, linear regression and correlation analysis indicates that soil fractal dimensions have a highly significant correlation with soil properties (*p*<0.01). Estimation of soil fractal dimension under different BSCs is helpful in determining changes in soil properties and vulnerability of desertification. More specifically, lower values indicate fine particles removal in soil coarsening process due to wind erosion in desertification-vulnerable regions. With the loss of fine particles, SOC and soil nutrients decreased concomitantly. Accordingly, higher *D* values suggest sand particles disintegration, fine particles accumulation and soil properties recovery as affected by BSCs. Furthermore, *D* values indicate the potential of desertification as well. The higher *D* values implies the greater possibility of wind erosion due to the bigger proportion of fine particles. Once the sandland loses the protection of BSCs or the actual wind velocity exceeds the threshold, accumulative fine particles will be eroded by destructive wind in a short term. Therefore, fractal dimension is sensitive and useful index for quantifying soil properties variations in the evolution of a desert, and significantly implies for soil degradation and desertification induced by wind erosion.

## Conclusions

In the Mu Us desert, the establishment and succession of BSCs caused positive changes in topsoil properties. There was a clear tendency to increase from sandland to BSCs sites in soil total porosity, SOC, soil nutrients, clay and silt particle contents, accompanying decrease in soil bulk density, and sand particle contents. With the succession of BSCs, significant difference occurred between mosses crust and uncovered sandland. Linear regression and correlation analysis showed that *D* values had significant linear relationships with soil bulk density, total porosity, SOC, soil nutrients (except K_T_) as well as soil PSD with R^2^ values ranging from 0.494 to 0.955 (*p*<0.01). Fractal dimension is sensitive to soil coarsening and nutrients losses process in the evolution of desertification, and it is now proven to be a sensitive and practical index for quantifying changes in soil properties and additionally implies desertification and vulnerability to desertification.
